# Knowledge, perceptions, and management of symptoms of hormonal imbalance among adolescent girls in selected schools in Ghana: a qualitative exploratory study

**DOI:** 10.3389/frph.2024.1502352

**Published:** 2024-12-13

**Authors:** Sawudatu Zakariah-Akoto, Benjamin Abuaku, Godfred Egbi, Bismark Edem Kofi Klu, Eric Kyei-Baafour, Michael Fokuo Ofori, Collins Stephen Ahorlu, Dorothy Yeboah-Manu

**Affiliations:** ^1^ Department of Nutrition, Noguchi Memorial Institute for Medical Research, University of Ghana, Accra, Ghana; ^2^ Department of Epidemiology, Noguchi Memorial Institute for Medical Research, University of Ghana, Accra, Ghana; ^3^ Department of Immunology, Noguchi Memorial Institute for Medical Research, University of Ghana, Accra, Ghana; ^4^ Department of Bacteriology, Noguchi Memorial Institute for Medical Research, University of Ghana, Accra, Ghana

**Keywords:** knowledge, perceptions, management, symptoms of hormonal imbalance, adolescent girls, Ghana

## Abstract

**Introduction:**

Adolescent girls are more sensitive to hormonal imbalance with major impact on their nutritional, reproductive, physical, psychosocial, and academic wellbeing. This study explored adolescent girls’ knowledge and perceptions of causes and management of symptoms of hormonal imbalance.

**Materials and methods:**

Using a qualitative approach, focus group discussions were conducted with 116 assented in-school adolescent girls aged 10–19 years between 3rd and 19th October 2022. Two urban and two rural communities in two regions were purposively selected for the study. Using a convenient sampling approach, participants were recruited from Upper Primary, Junior, and Senior High schools. Data was analyzed thematically using both inductive and deductive approaches.

**Results:**

Senior High School participants had a fair understanding of hormonal imbalance in both regions. Symptoms were perceived to include headaches, heavy and painful periods, and pimples, most of which participants alluded to experiencing. Perceived causes included natural process of adolescent growth, consumption of unhealthy diets, use of cosmetics and medications. Perceived reproductive effects included infertility, pregnancy disorders, breastfeeding challenges, and low sexual drive. Physiological effects included extreme fatigue, loss of appetite and impaired growth. Psychosocial and academic effects included mood swings/irritability, low self-esteem, poor inter-personal relationship, poor concentration in class and irregular school attendance. Female relatives and friends were mostly consulted for advice. Self-medication, dietary modification, physical activities, and personal hygiene were adopted to manage perceived symptoms. Self-medication was common, but physical activities and dietary modification were also adopted by most participants to manage symptoms.

**Conclusion:**

It is recommended that Ghana Education Service should formally incorporate hormonal-related issues into its School Health Education Programmes to enhance knowledge, attitudes, and management among adolescent girls at all levels of education.

## Introduction

Adolescence (age 10–19 years) is a period of transition from childhood to adulthood through a myriad of physical, psychological and social developmental changes (1). These developments are regulated by changes in the levels of hormones—chemicals produced in the body that function as important regulators of bodily processes (2). Hormones are created by the endocrine glands—located in every part of the body—and transported through the bloodstream to different tissues and organs of the body, basically taking charge in regulating every major system (3). Over 50 hormones regulate key bodily functions, with primary ones like estrogen, progesterone, insulin, and androgen, playing crucial roles in reproductive, metabolic, and growth processes (3, 4). There is also evidence showing that behavioural factors such as depression and anxiety disorders including diminished interests and impaired cognition, sleep and appetite, which are reported to be more common particularly among menstruating women, are driven by fluctuating sex hormones (5). Knowledge of these hormonal activities could, therefore, be an important strategy for managing women's health and well-being.

Production of hormones in their non-optimal proportions may result in hormonal imbalance (3, 6). Hormonal imbalance is prevalent at different times of the female reproductive cycle especially during menstruation, pregnancy, and menopause. Women in their reproductive age, including adolescent girls, are most susceptible to hormonal imbalances with an estimated prevalence of 30% (2). Clinically, hormonal levels must be in excess or deficit from the normal range expected for one to be considered as experiencing hormonal imbalance. Serum hormonal analysis is, therefore, critical in determining hormonal imbalances in populations rather than basing diagnosis solely on symptoms.

Hormonal imbalances may have adverse impact on the physical, reproductive, psychosocial, and general health and well-being of adolescent girls, including their major role in controlling appetite/eating disorders, weight gain/loss, menstrual-related disorders, poor concentration, and reduced interests in activities (2, 7, 8). Depending on which specific hormone is affected, different signs and symptoms may be presented (3). Fluctuations in the levels of androgen and estrogen, for instance, have been linked to painful menstruation, bloating, weight gain, headaches, backpain, cravings for unhealthy foods and allergic reactions (2, 9). Insufficient production of insulin results in elevated blood sugar/glucose levels and may cause obesity and Type 2 diabetes (2). The strong influence of hormonal imbalance on adolescent girls' health, therefore, makes it an issue of serious public health concern that needs urgent attention (1).

There is substantial knowledge gap and misconceptions about menstruation- a product of hormonal activity- among adolescents. The situation leads to fear and anxiety during menstrual periods (1). Many studies have focused on hormonal imbalance and infertility-related issues among women, hormonal profile of and concentration in adolescent girls and psychological effects of polycystic ovary syndrome (10–12). In Ghana, studies have focused on infertility among women, menstruation-related issues, and uptake of hormonal contraceptive among adolescent girls (10, 13–15). Studies on hormonal imbalance in general are scanty despite its potential effects on adolescent girls' general health outcomes. The studies have concentrated on the relationship between hormonal imbalance and reproductive health among adolescent girls from external perspective without attention to girls' perspective.

Poor diet and eating disorders are identified among the key influencers/drivers of hormonal imbalance. On the contrary, eating healthy diets is identified as one of the main lifestyle changes for addressing hormonal imbalance (2, 8, 16, 17). This study aims to address the knowledge gap by exploring adolescent girls’ understanding of hormonal imbalance, its dietary influences, and their personal experiences with its symptoms. Here, we report findings on their knowledge, perceptions, and self-management strategies for symptoms of hormonal imbalance.

## Materials and methods

### Objective of study

The objective of the study was to explore adolescent girls' knowledge, perceptions, experiences, and management of perceived symptoms of hormonal imbalance.

### Study design

The study employed a qualitative approach via focus group discussions (FGDs) to explore and understand adolescent girls' knowledge and perceptions about hormones and hormonal imbalance as they relate to their reproductive and psychosocial health. FGDs were chosen on grounds of convenience and economy of time. Participants felt relaxed to give information in homogeneous groups but might feel ill at ease if alone with the researcher to give sensitive information on their reproductive health. Using FGDs facilitated fast data collection and minimised disruption of normal school activities for the girls. The method further facilitated in-depth understanding of participants' knowledge and perceptions of hormonal balance and imbalance and how these relate to their health and well-being.

### Selection of study locations and target population

Greater Accra and Northern Regions were purposively selected to represent the southern and northern sectors of the country respectively. They were also selected based on their peculiar dietary dietary practices, which are partly driven by their peculiar agro-ecological differences/locations. The southern sector is characterized by thick rainforest compared with the north, which is predominantly grassland, dry climate, and relatively higher temperatures; thus, influencing the kinds of crops and animal-source foods produced in the regions. These agro-ecological differences have implications for their dietary practices. Peculiar dietary practices that may influence hormonal imbalance include the variety, quality and frequency of consumption as well as gender-based food preference, taboos and restrictions.

In each region, a district was selected based on the availability of at least two senior high schools—one in a predominantly urban setting and the other in a predominantly rural setting. La Nkwatanang Municipality in Greater Accra Region (GAR) and Tamale Metropolis in Northern Region (NR) were selected. The study communities were: Adenta and Damfa in La Nkwantanang Municipality and Wulshei-Kukuo and Banvim in Tamale Metropolis. In each community, three schools were selected: upper primary (UP), junior high school (JHS) and senior high school (SHS) -either co-educational or only girls' schools.

Adolescent girls aged 10 to 19 years who had experienced menarche and had been having their menstrual periods for the past one year were included in the study. This target population ensured that girls in upper primary, junior, and senior secondary schools were given equal chances of being enrolled into the study. Girls in this age-group have also been reported to experience symptoms of hormonal imbalance and its associated problems more frequently than those in other age-groups (2). Out-of-school adolescent girls and those who could not read were excluded from participating in the study because of issues related to ease of organizing them as well as getting informed consent from their parents/guardians prior to data collection. It was, on the contrary more convenient to focus on in-school adolescent girls using their heads of schools and teachers to organize them including acquiring informed consent of parents/guardians. Translating the concept “hormonal imbalance” into the local vernacular for out-of-school adolescent girls and those who could not read posed another challenge to the researchers. Adolescent girls in lower primary, those less than 10 years old and those who had not experienced menarche were also excluded.

### Data collection

Twelve FGDs were held with a total of 116 participants between 3rd and 19th October 2022 in the two selected municipalities according to the schedule specified by Ghana Education Service (GES) Headquarters. In each municipality, six FGDs—three per selected community (or school)—were held with an average of 9 participants. A summary of the distribution of FGDs and participant groups is presented in Table 1.

**Table 1:** Summary of FGDs and participant groups.

Participant categories	Northern region (Tamale Metropolis)		Greater accra region (La Nkwantanan Municipality)		Total FGDs per educational level	No. of participants
	Rural	Urban	Rural	Urban		
Upper Primary (UP)	1 (10)	1 (10)	1 (6)	1 (10)	4	36
Junior High Sch. (JHS)	1 (10)	1 (10)	1 (9)	1 (10)	4	39
Senior High Sch. (SHS)	1 (10)	1 (10)	1 (10)	1 (11)	4	41
**Total FGDs & participants**	**3** (**30)**	**3** (**30)**	**3** (**25)**	**3** (**31)**	**12**	**116**

Prior to the FGDs, the FGD guide was pretested at University of Ghana Basic School, located in a different municipality in Greater Accra Region. Pre-testing helped to assess the appropriateness of the questions (ease of understanding), relevance (to the study objectives) and minimum time required to complete a focus group discussion session.

School teachers facilitated the selection of participants for the study. FGDs explored issues such as participants' socio-demographic backgrounds and those of their parents/guardians, their knowledge and perceptions about hormones in general and hormonal imbalance in particular in respect of its symptoms, causes, effects, and management. Additionally, participants' lived physiological experiences (including painful and irregular menstruation, muscular cramps, heavy bleeding, and acne) and psychosocial experiences (including irritability, restlessness, fatigue, mood swings, anger. and depression) were explored.

Initial face-to-face contacts and phone calls with selected schools and participants were used to agree on dates convenient to them for the FGDs in their schools. The discussions were conducted in English with occasional explanations and prompting in Twi and Dagbani. Each FGD lasted about one and a half hours.

### Data coding, management, and analysis

A note-taker assisted the moderator to record all sessions of the discussions. Notes taken during discussions were reviewed and coded to identify emerging themes and issues in need of follow-up and to determine data saturation. The review indicated that data saturation was attained for each of the issues investigated by the sixth FGD in each municipality. In other words, no additional themes or ideas emerged from reviewing the sixth FGD transcript in either municipality. All recorded discussions were transcribed verbatim into English by a team of research assistants who were trained on the project and were familiar with transcribing qualitative data. A codebook, informed by *a priori* themes, the interview guide, and emerging themes from a few transcripts, was developed to guide the coding process to achieve uniformity. All the transcripts were coded by two members of the research team and reviewed by a third member.

Using the thematic approach, transcripts were thoroughly read for familiarization to determine patterns in the responses (18, 19). Following this, all expressions were systematically coded across all transcripts. Similar codes were then collated under their appropriate themes and sub-themes to facilitate analysis of data guided by consensus/agreement as against conflicts/disagreements related to two main variables: location (region, rural/urban) and education (upper primary, junior high school and senior high school). Verbatim quotations depicting sub-themes were used to support findings. A summary of the final four themes and their respective sub-themes is presented in Table 2.

**Table 2:** Themes and sub-themes.

Theme	Sub-theme
Menstrual pattern among participants	*
Knowledge, perceptions, symptoms and causes of hormonal imbalance	Knowledge of hormones and hormonal imbalance
	Perceived symptoms of hormonal imbalance and participants’ experiences
	Perceived causes of hormonal imbalance in adolescent girls
Perceived effects of hormonal imbalance in adolescent girls	Reproductive health
	Physiological health
	Psychological health
	Academic performance
	Social interaction
Management of perceived symptoms of hormonal imbalance in adolescent girls	Persons and places consulted for management of symptoms
	Strategies adopted to manage perceived symptoms

* No sub-theme emerged for analysis.

### Ethical considerations

Study protocol was approved by Institutional Review Board of Noguchi Memorial Institute for Medical Research, University of Ghana, Legon (Reference Number: CPN 053/21-22). Permission to undertake the study in selected schools was granted by Ghana Education Service (GES). After giving official approval, GES Headquarters notified the two Regional Directorates and their respective municipal directorates of the study with letters of approval. Informed verbal consent was then obtained from the heads of the selected schools; however, all participants and their parents/guardians gave written consent prior to the FGDs. Regarding the safety of participants and the research team, all Ghana Health Service (GHS) COVID-19 protocols were duly adhered to including providing facemasks and sanitizers to all participants and using open spaces suggested by school authorities for all FDGs. Additionally, all FGD moderators and note-takers received sensitivity training to ensure that they are sympathetic towards the needs and fears of the adolescent girls.

## Results

### Participants' socio-demographic characteristics

Participants' socio-demographic characteristics including the educational and employment statuses of their parents or caregivers are presented in Table 3.

**Table 3:** Participants’ and parents’ socio-demographic characteristics.

Variables	(*N* = 116)	(%)
Region		
Northern	60	51.8
Greater Accra	56	48.2
Age (years)		
12–14	41	35.3
15–19	75	64.7
Education		
Upper Primary	37	31.8
JHS	39	33.6
SHS	40	34.5
Place of residence		
Urban	61	52.6
Rural	55	47.4
Religion		
Christian	65	56.0
Muslim	51	44.0
Father's education (formal)		
None	17	14.7
Basic	22	19.0
SHS	32	27.6
Tertiary	25	21.5
Don’t know	20	17.2
Father's employment status		
Employed	104	89.7
Unemployed	1	0.9
Don’t know	11	9.4
Father's place of work		
Formal	31	26.7
Informal	72	62.1
Don’t know	13	11.2
Mother's education (formal)		
None	31	26.7
Basic	36	31.0
SHS	26	22.4
Tertiary	10	8.6
Don’t know	13	11.2
Mother's employment status		
Employed	109	94.0
Unemployed	4	3.4
Don’t know	3	2.6
Mother's place of work		
Formal	18	15.5
Informal	94	81.0
Don’t know	4	3.4

Participants in the study were aged 12–19 years with the majority (64.7%) in the older adolescent age-group (15 to 19 years). Almost equal proportions of participants were drawn from the three educational levels: UP (31.8%), JHS (33.6%) and SHS (34.5%). There were slightly more urban participants (52.6%) than rural (47.4%), and the majority (56%) of participants were Christians.

Most participants' parents had some level of education. Most participants' fathers (27.6%) had SHS as the highest level of education attained whilst majority of mothers (31%) had basic as the highest level of education attained. Most parents were employed (89.7% of fathers and 94% of mothers) and in the informal sector (62.1% of fathers and 81% of mothers).

### Menstrual pattern among participants

By recall, participants provided data on their age at menarche, menstrual cycle, regularity of their cycle and duration of menstruation (Table 4).

**Table 4:** Menstrual pattern.

Category	Freq.	%
Age at Menarche (years)		
<10	1	0.9
10–11	17	14.7
12–13	70	60.3
14–15	27	23.2
>15	1	0.9
**Total**	**116**	**100**.**0**
Monthly menstruation		
Regular (every month)	103	88.8
Irregular (not every month)	13	11.2
**Total**	**116**	**100**.**0**
Menstrual cycle (Days)		
20	4	3.9
21–35	99	96.1
**Total**	**103**	
Duration of menstrual flow (days)		
2–3	1	0.9
3–7	113	97.4
7–8	2	1.7
**Total**	**116**	**100**.**0**

Most participants (70/116–60.3%) experienced menarche between the ages of 12–13 years. Most participants (103/116–88.8%) reported regular monthly menstruation. Of the 103 participants, 99 (96.1%) had menstrual cycles of between 21 and 35 days. Almost all participants (113/116–97.4%) had menstrual flow lasting between 3 and 7 days.

### Participants' knowledge and their perceived symptoms and causes of hormonal imbalance

Three sub-themes emerged under this theme, namely knowledge of hormones and hormonal imbalance, perceived symptoms of hormonal imbalance and participants' experiences and perceived causes of hormonal imbalance in adolescent girls.

#### Knowledge of hormones and hormonal imbalance

The most common understanding of hormones was that they were body chemical substances or particles that cause changes in the physical growth and development of both external and internal organs including height, weight, breasts, and hips. Hormones were also described as chemicals that targeted specific internal organs including the heart, lungs, and liver for optimal performance, aided in digestion, influenced menstruation, and caused one to feel pain:

“They (hormones) cause physical changes such as increase in height and weight and development of parts of our body like the breasts and hips” (SHS, Urban, NR).

Additionally, most urban SHS participants were aware of the different kinds of hormones, particularly estrogen, progesterone, testosterone, prolactin, and thyroxine. A participant explained the functions of specific hormones she was aware of:

“Prolactin in females helps in the production of breast milk after birth. Testosterone enables the production of sperm in males. Thyroxine prepares the body for emergencies” (SHS, Urban, NR).

Most participants in SHS in both regions spontaneously described hormones in terms of their focus—parity, quantity, stability, functionality, and appropriateness without prompting. Regarding parity, participants indicated that an adolescent girl could experience symptoms of hormonal imbalance if she had more of the male than female hormones, which may lead to her exhibiting masculine features:

“Hormonal imbalance means they aren’t equal; for example, as a female you’re supposed to have more estrogen than testosterone. So, if you’ve more testosterone than estrogen, it means you’ve a problem” (SHS, Urban, GAR).

“A woman told us earlier this morning that certain ladies unnaturally have beards. That means they’ve more of the male hormone in their bodies so they could go to hospital to get the hormones regulated” (SHS, Rural, GAR).

There was consensus among SHS participants in both regions that hormonal imbalance occurred when the hormones in the body were not in the right proportions or were either too much or too little for the body to function optimally:

“It means the system lacks some hormones while others are excessive” (JHS, Urban, GAR).

“That person hasn’t the right number of hormones in the body. For instance, if she's supposed to have three hormones but has only one, then she doesn’t have the right number. That's what we know about hormonal imbalance” (SHS, Urban, NR).

“Something that's more than required. It means it isn’t in its required proportion” (SHS, Urban, GAR).

They further described hormonal imbalance in terms of the stability of their activities or production in the body:

“Hormonal imbalance is a condition in which the activity of the hormones in the body doesn’t remain constant including their production in the body” (SHS, Urban, NR).

Participants perceived improper functioning of hormones as imbalance:

“It means that the hormones in the adolescent don't function well” (SHS, Rural, NR).

Some UP participants initially found the concept incomprehensible. Prompts by other participants aided their awareness and identification with the symptoms.

Participants indicated six main sources of information about hormones. School lessons including Social Studies and Integrated Science were the foremost sources of information among all groups. Extra or vacation classes in other schools were additional sources for participants in urban Northern JHS. Related literature, the internet, friends. and healthcare providers were other popular sources for most SHS participants. JHS participants identified church programmes as their other source of information.

#### Perceived symptoms of hormonal imbalance and participants' experiences

Participants across all groups were knowledgeable about the symptoms of hormonal imbalance and indicated symptoms adolescent girls in general may experience including headaches/migraines, pimples, irregular, heavy, prolonged or missed periods, pains in the waist and breast during menstruation, general bodily pains and weakness, emotional slump/mood swings and, in extreme cases, depression. Less popular symptoms among adolescent girls in general were hair on chest, sweaty skin and hair and weight loss. Some participants indicated that they experienced similar symptoms, and the foremost symptoms were menstrual cramps/pains (84/116–72%), headaches/migraine (51/116–44%), heavy periods (33/116–28%), pimples/acne (42/116–36%), constipation (28/116–24%), irregular periods (13/116–11.2%), hot flashes (9/116–7.7%), and breast tenderness (11/116–9.4%). Less common symptoms experienced by participants were waist pains (5/116–4%), vaginal dryness and itchiness (4/116–3.4%), sweaty skin (2/116–1.7%) loss of hair (1/116–0.8%), and night sweats (1/116–0.8%).

There were no marked regional variations in the symptoms reported by participants except in the case of headaches/migraines, acne/pimples and constipation which were more frequently reported in Northern Region. Loss of hair and night sweats were reported in only Greater Accra while waist pains and irregular periods were reported in Northern Region. Irregular periods were experienced frequently among UP and Northern Region participants. Bad body odour (6/116–5%) was reported by few UP and JHS participants in both regions. Participants typically experienced symptoms either just before or during their periods. Persistent headaches, cramps, pimples/acne, irritability, nausea, and other symptoms were more frequently experienced just before periods. Pimples/acne was the main symptom experienced after periods.

#### Perceived causes of hormonal imbalance in adolescent girls

Participants in both regions attributed symptoms of hormonal imbalance to a wide range of causes belonging to two main perceived categories. The first comprised inevitable natural symptoms of adolescence. This perception was held particularly of pimples/acne among all participants:

“Some people also say that when you get to your adolescent stage, that's when the pimples come” (UP, Rural, GAR).

“Madam, my teacher told me that it's a physical change with an adolescent girl or boy so when you’re an adolescent, you get those changes. Some people go through them some don’t” (JHS, Urban, NR).

The second category of causes belonged to lifestyle choices. Participants were unanimous about the effects of their dietary habits, use of body products/cosmetics and medication on the symptoms they experienced. The relevant dietary practices included consumption of diets rich in fats and oil, sugar-sweetened beverages, sweets (candies), milk, egg and bouillon cube (magi cube) and drinking cold water:

“Some said when you eat oily foods, the pimples will start to come” (UP, Rural, GAR).

“I think eating too many sweets can cause menstrual cramps because if you eat sweets too much and it's time for you to menstruate, it can cause abdominal pains and can also affect your menstrual cycle” (SHS, Urban, GAR).

“Drinking cold water gives headaches and prolongs your duration of menstruating”. (SHS, Urban, NR).

All UP participants in one rural location and a few from the other groups, for instance, perceived eating of groundnut as a cause of pimples/acne. Consumption of milk and egg was linked with heavy periods by some SHS participants in urban Northern Region. Use of condiments such as bouillon cubes in food was linked to waist and menstrual pains by a few JHS participants in rural Northern Region. The following quotations buttress these findings:

*“*Some people have been saying that when you eat groundnut, the pimples come” (UP, Rural, GAR).

“As for me, if I take milk and egg, it (menses) comes more so I don't take egg and milk” (SHS, Urban, NR).

“Madam too much magi can cause waist pains. It can cause the stomach to hurt as well” (JHS, Rural, NR).

Using body creams and some medications was perceived to cause pimples and sweaty skin in both regions:

“Please, I use a pomade called “cocoderm” and it causes pimples to appear on my face” (UP, Urban, GAR).

“Pomade made pimples come and they said I’ve oily face” (SHS, Rural, NR).

Some participants perceived that stress, inadequate sleep, and noise caused headaches. They also believed that constipation could trigger prolonged menstruation and headaches. All rural JHS participants perceived heavy periods to be results of having excessive blood:

“I thought that if one has a lot of blood, the flow will be heavy. I think my flow is heavy because I’ve a lot of blood” (JHS, Rural, GAR).

### Perceived effects of hormonal imbalance in adolescent girls

Five sets of perceived effects of hormonal imbalance in adolescent girls identified by participants included effects on reproductive, physiological, and psychological health. The others were effects on academic performance and social interaction.

#### Reproductive health

Some UP and JHS participants in one urban location perceived that the symptoms had no effect on the girls' reproductive health. They reported that irregular menstruation would not affect one's ability to have children in future. It was, however, mutually reported among all the five participant groups that infection of the womb may cause infertility and the most frequently reported infection was fibroids.

“You may have some problem with producing babies if your menstruation doesn’t come regularly, I think it can cause fibroids” (JHS, Urban, NR).

The other mutual perceptions were low and high libido usually after menstruation. Inability to effectively breastfeed through lack of prolactin and pregnancy disorders were other less commonly mentioned effects of hormonal imbalance on reproductive health.

#### Physiological health

Sleeplessness was identified as an effect of the symptoms of hormonal imbalance on the physiological health of adolescent girls by all the five groups especially SHS participants in one urban location. Extreme fatigue was experienced by all SHS participants in one urban location in Northern Region. The same effect—fatigue—was experienced by the minority of participants in rural Greater Accra Region. Other less common physiological effects included loss of appetite, stomach ulcer (long term effect of loss of appetite), impaired growth, poor vision, dry and wrinkled skin, and skin rashes.

#### Psychological health

Irritability was perceived to be a psychological effect of hormonal imbalance by most SHS and JHS participants but by few UP participants. Diminished interest in activities affected all SHS participants. Sadness, dullness and feeling of insecurity/fear/apprehension affected both rural and urban participants at all the three educational levels. Mood swings, forgetfulness, low self-esteem, and timidity were other effects mentioned by some participants across the three educational levels and urban locations in both regions. A participant said:

“I think during menstruation, mood swings occur so I think the hormones in us bring about all these. Sometimes you’ll feel angry, stressed, and weak. So, I think it's all caused by hormones”. (SHS, Rural, GAR).

#### Academic performance

Poor concentration and distraction in class was an academic effect experienced by most participants in all the five groups. Missing classes through irregular school attendance—leading to poor academic performance—as well as inability to think straight was also mentioned by some participants across the five participant groups:

“Sometimes if you feel pains, the teacher may be talking but you’ll be absentminded” (JHS, Rural, NR).

“When they’re teaching and you’ve headache, you can't think straight. You can’t concentrate” (JHS, Rural, GAR).

#### Social interaction

Poor interpersonal relationships affected most participants in both regions (7/12 FDGs). Additionally, hormonal imbalance caused few SHS and JHS participants to be isolated from peers at school and home:

“When you’re sad, your friends move away from you. Later they’ll come back to you and say, “you’d frowned”. But sometimes you wouldn't be aware that you just did that, but because of the hormone in your body, you may have done that but won’t be aware” (SHS, Urban NR).

“Sometimes you can be sitting and suddenly you don't even know what's making you angry, your friend would come and be talking to you, but you won't even mind her. She would also be angry and leave you” (SHS, Urban NR).

### Management of perceived symptoms of hormonal imbalance among adolescent girls

Generally, participants were comfortable talking about their experiences in managing their symptoms. They discussed persons and places they usually went to consult regarding the management of their symptoms. The strategies used to manage the symptoms and their perceived effectiveness are described under the following sub-headings:

#### Persons and places consulted for management of perceived symptoms

Participants consulted two main places for the management of their symptoms. The first place was the chemical shop/pharmacy which was mostly patronized by rural JHS and SHS participants. Urban SHS participants consulted school health officers. Consensus among most participants was that female relations, particularly mothers, sisters, and grandmothers, were the dominant persons consulted for guidance to manage their symptoms. According to SHS participants, they consulted females rather than males, including fathers, partially because they felt either shy or afraid to tell the latter about the symptoms.

Almost all UP participants would consult their mothers or sisters for advice on their symptoms. A few SHS participants in both rural and urban locations would rather consult their fathers especially when they needed money to buy sanitary pads during their periods. The participants explained that it was easier to get fathers than mothers to fund their sanitary pads. In some cases, mothers would ask the adolescents to go to their fathers for money needed to buy sanitary pads. A SHS participant explained why she would go to her father rather than her mother:

“Frankly speaking, I always talk to my father. If I’m menstruating and need pads for instance, and I ask my mother, she would tell me to go to my father, and it's he who would give me the money. So why tell her?” (SHS, Urban, NR).

Some participants were also of the view that fathers supported mothers to acquire adolescent sanitary pads:

“My mum takes the money from my father and buys them for me” (SHS, Urban, GAR).

Consulting books, drugstore dealers, teachers, health prefects, school health officers/worker and trusted friends were options for SHS and JHS participants. Boarding SHS participants explained that they usually consulted teachers, headteachers, friends and school health workers when in school but once they came home, they consulted parents.

A few urban SHS and rural JHS participants indicated they kept their symptoms to themselves. One of them explained her habit of non-interaction and non-communication with her family members about her reproductive health issues:

“Because I’m staying with my sister and I never sit to talk about such things with her so it's always difficult to talk about them with her” (JHS, Rural, NR).

#### Strategies adopted to manage perceived symptoms of hormonal imbalance

Participants discussed the strategies they adopted mostly in the management of headaches/migraines and menstrual pains/cramp—two of the most frequent symptoms reported. Few participants indicated actions adopted to manage body odor, heavy periods, and vaginal dryness/itchiness. Management of prolonged, missed, and irregular periods, breast tenderness, pimples, constipation, fatigue, sweaty skin, night sweats, hot flashes and other symptoms was hardly discussed.

Participants used both medical and non-medical lifestyle-related strategies to manage their symptoms. Medications used to relieve headaches, waist pains, abdominal cramps and other symptoms consisted mainly of pain killers such as paracetamol and brufen. Others were antibiotics (e.g., amoxicillin), antacid (e.g., magnesium trisilicate and aluminum hydroxide), and folic acid.

Some participants in UP and JHS indicated their mothers would usually give them the medicines to take but this practice appeared to be uncommon among SHS participants.

“My mother gives me medicine because I'm unable to sleep or eat. I throw up during menstruation” (UP, Rural, GAR).

Most SHS participants, however, indicated that they took pain killers when they experienced pains especially during menstruation. One participant clearly stated:

“I take pain killers like “Escape pain” to reduce the menstrual pain” (SHS, Rural, NR).

The other medical-related management strategies were consulting a pharmacy or drugstore for assistance, going to a facility and consulting school health authorities. Participants who visited a health facility or consulted a doctor were usually those with extreme menstrual pains. A participant narrated her experience with menstrual cramps and how it was managed:

“The menstrual pains hurts so much that I cry and sometimes go to hospital with my mother and the doctor prescribes brufen” (JHS, Rural NR).

A summary of strategies adopted in managing participants' symptoms in relation to pain is presented in Table 5.

**Table 5:** Strategies used to manage symptoms experienced by adolescent girls.

Medical -related strategies	Lifestyle behaviour			
	Physical activity	Personal hygiene	Dietary modification	Others
Taking medicines	Regular exercises	Applying lime, water & ash or alum to the armpit or vagina	Eating healthy Balanced diet including plenty fruits and vegetables	Resting
Consulting a pharmacist/chemical seller	Being active all the time	Frequent daily baths and changing pads	Avoiding sugar-sweetened foods including toffee, ice-cream, and powdered milk	Massaging affected parts of the body with warm water
Regular hospital check-ups and seeking doctors’ diagnoses			Drinking water (cold hot)	Not carrying heavy loads
Consulting school health nurses and prefects in charge of sick bays			Eating spicy foods—to manage nausea	Applying hot compression on abdomen
				Enduring pain
				Taking herbal concoctions

The non-medical or lifestyle-related strategies included physical activities, maintaining good personal hygiene and dietary modification. Other management strategies included sleeping/resting, massaging affected parts with hot water/using hot-water bottles or applying hot compression to the lower abdomen, avoiding heavy loads on the head, taking herbal concoctions and enduring pain.

Physical activities mentioned by participants were regular exercises like jogging and being always active. Actions participants took to ensure personal hygiene included regular daily baths and changing of their sanitary pads. Participants who indicated experiencing body odour mentioned applying and cleaning their affected body parts such as the armpits and spots between the thighs with lime, ash, and alum.

Dietary modifications adopted by participants were mainly eating balanced diets including lots of vegetables and fruits, reduction in the intake of sugar-sweetened foods and drinking lots of water (hot or cold, depending on individual differences) during menstruation. Participants who experienced nausea managed it by eating spicy foods. One urban JHS and UP participant each in Northern Region, indicated managing menstrual pain with traditional or herbal medicines:

“Sometimes too my mother would boil some herbs and I take them” (JHS, Urban, NR).

“They give me “dagbantim (herbal medicine)”” (UP, Urban, NR).

Very few SHS and JHS participants mentioned how they managed heavy menstruation and vaginal dryness and itchiness. A SHS participant mentioned taking hot tea to manage heavy menstruation:

“I know some friends who take hot tea to stop the menses from coming heavily” (SHS, Urban NR).

An urban JHS participant indicated applying hot (minty) creams to the vagina to stop or reduce vaginal itches:

“When I'm talking to someone and the vaginal itching comes, I'll stand there and begin moving my legs to rub against my vagina. Sometimes if I’ve some Robb (minty-like cream), I'll apply it to my vagina so that I'll feel pains rather than the itches and that will prevent me from touching the affected spot” (JHS, Urban, GAR).

Participants assessed as generally effective the strategies for managing symptoms. Those who found the medical strategies ineffective may resort to activities perceived to divert their attention from their pains, including reading and sleeping.

## Discussion

The study explored knowledge, perceptions and symptoms of hormonal imbalance among in-school adolescent girls in Greater Accra and Northern regions of Ghana. It further explored symptoms they experienced and how they were managed. Four key themes that emerged from the findings are discussed.

### Menstrual pattern among participants

The average age at menarche was 12.7 years with more than half (70/116) of the participants experiencing menarche between the ages of 12 and 13 years. This finding is consistent with findings from previous studies (20–25); however, in two other studies conducted among tertiary female students aged 20–25 years in Northern Region and among women aged 10–49 years in Ashanti Region, both in Ghana, age at menarche was found to be higher by approximately one year (13.7 and 14 years respectively) (14, 26). The marked differences found between these studies and the current study could be explained by differences in generational maturity. Older generations experienced menarche at a much older age than younger generations. As far back as 1995, a study reported that globally, the age of menarche had seen a downward trend from age 16.5 years in the 1840s to 13 years at the time of the study (27). It is the view of this study that if current conditions remain constant, the age at menarche may continue to drop with implications for the general well-being of the adolescent girls. Regular menstruation, that is, discharge of blood from the uterus at regular monthly intervals (28), was reported by most participants (89%) with only 11% reporting irregularities in their menstruation. Irregularities in menstruation reported in the current study included experiencing menstrual cycle only once since menarche, having menstrual flow once in several months or never knowing when their next menstrual flow would occur. The prevalence (11%) of these irregularities was, however, low compared with those found in other studies (24, 25). Plausible reasons for irregularities in menstruation include age at menarche as reported in a similar study in Portugal (24).

This study found that most (99/103–96%) participants experienced normal menstrual cycle—regular shedding of blood from the uterus every 21–35 days. This finding is consistent with that from earlier studies (24, 25). Unlike other studies, however, this study did not report cases of menstrual cycles of over 35 days (22). Experts on adolescent health issues explain that 90% of the menstrual cycle in adolescence will be within the 21–45 days cycle even though, at the initial stages of menstruation, some adolescents may experience shorter cycles of under 20 days and longer cycles of over 45 days. Shorter menstrual cycles of 20 days or less, according to the experts, are expected to normalize by the third year after menarche to a cycle of 21–35 days as experienced in adult women. The most common duration of menstrual flow during each menstrual cycle was 3–7days among participants. This finding compares well with findings from earlier studies (22, 29). Potential factors for the relatively low prevalence of menstrual irregularities may include adopting healthier lifestyle behaviour such as engaging in physical activities and avoiding perceived unhealthy dietary practices. Additionally, all participants indicated they were healthy at the time of data collection. Participants’ sources of information about hormones including the school, healthcare professionals and church programmes may have also contributed to improved knowledge on health-related issues and, hence, their healthy lifestyle behaviour posibly accounting for the low prevalance of menstrual irregularities among them.

Several drivers could explain the fall in the age at menarche including improved health, well-being, and nutrition. Other drivers may include personal factors such as genetics, physical activity levels and nutritional status; the food environment and socio-economic factors (27, 30). Implications of the fall in the age at menarche could range from increased teenage pregnancy to increased risk of developing breast cancer in later life, diabetes and other non-communicable diseases (27).

### Knowledge, perceptions, symptoms and causes of hormonal imbalance

The identification of hormonal imbalance in relation to inadequacy, inappropriate, inefficient functioning of some hormones in the body aligns with the scientific definition of hormones (3, 7). This level of knowledge among some participants may be attributed to their educational curricula where aspects of adolescent growth and development are taught especially in the natural sciences and social studies. The internet and participants' ability to read may be other reasons for this high level of knowledge. Perceived symptoms of hormonal imbalance included cramps/pain, heavy periods, irregular menstrual cycles and prolonged periods with a prevalence of 11%–26% in adolescents reported elsewhere (23). Other perceived symptoms included headaches, pimples/acne, waist pain, breast tenderness, vaginal dryness, and itchiness. Emotional slump, mood swings, constipation and fatigue were also mentioned. These perceived symptoms align with what have been reported in the scientific literature as symptoms of hormonal imbalance expected not only in adolescent girls but in older women as well (2, 8, 9).

Almost all participants alluded to experiencing at least one of these symptoms—a finding that has been reported in several other studies (2, 23, 24, 28). The most reported symptoms experienced among adolescents in this study were menstrual cramps/pains, irregular menstrual cycles, headaches, and pimples/acne. Menstrual cramps are medically referred to as dysmenorrhoea- a crampy lower abdominal pain during menstruation reported in about 50%–90% menstruating adolescents globally (31). In Italy, the prevalence was estimated at 84.1% and early menarche and prolonged periods were found to be associated with it (32). Participants in the current study linked the consumption of sugar-sweetened foods and condiments used in cooking to menstrual cramps. Implications of menstrual cramps have been reported to include polycystic ovary syndrome (PCOS) which has a 6%–18% prevalence among adolescent girls globally even though not all females with menstrual cramps have PCOS. PCOS is linked to other health-related challenges including the possibility of developing Type 2 diabetes and infertility (2, 23).

The school, through its curricula, was the foremost source of information on hormones and related issues. Identification of the dominant role of the school resonates with earlier findings by Amaede and Garti (14). The finding in the current study, however, contradicts a study in Pakistan, in which close to 78% of students had never had any class lesson on hormonal-related topics such as menstruation (28). Perhaps, the differences in findings could be attributed to the different focuses of the two studies: while the current study focused on hormonal imbalance in general, the Pakistani study focused on menstrual-related information.

Health-related books, the internet, healthcare providers, churches and friends have been identified in earlier studies as usual sources of information on general reproductive health among adolescents (33). Exposure to the internet and other media sources has increased in recent times, possibly helping to fill the information gap relevant to adolescent growth and development. Elsewhere, it has been reported that about 93% of adolescents are users of internet services (34)—a great potential in accessing health information among young people. It is also commonplace in Ghana for healthcare providers to be invited to schools to give health talks especially on the platform of the School Health Education Programme run by Ghana Education Service. Churches deliver health information to large congregations including students especially during festive occasions (35). Even though mothers and other female relatives were not the main sources of information on hormonal-related information in this study, they were indicated as the group most consulted by adolescent girls for advice when experiencing painful menstrual-related symptoms. Several studies have, however, reported mothers and other female family members as the main sources of information especially on menstruation (14, 28). The importance of adolescent girls' sources of health information derives from the status of adolescents as active consumers of information. Their sources of information must, therefore, be credible otherwise they stand a greater risk of experiencing adverse health outcomes.

### Perceived effects of hormonal imbalance in adolescent girls

Even though irregular menstrual cycles and prolonged periods (over 7 days) (36) were uncommon, participants were worried about the implications on their reproductive and psychosocial health and academic performance. Studies have, however, shown that these menstrual-related symptoms were to be expected especially among post-menarche adolescent girls who were between 12 and 13 years old (36). Headaches/migraine, neurovascular disorders that came second on the schedule of symptoms among participants, are rated by many medical-related studies among the most common disorders of the nervous system. They have a prevalence of between 15 and 18% globally—and affect women including adolescent girls, occurring about 3–4 times more than it occurs in men (37). Partly driving this disorder are fluctuations in the production of the sex hormones—estrogen and progesterone (37, 38). The finding that younger adolescent girls experience headaches frequently corroborates with previous findings (38). In addition to inflicting physical pain and disabling effects on school attendance and academic performance, headaches have also been associated with reduced quality of life and increased financial cost (37). Headaches among adolescent girls are issues of public health concern partly because they are reported to be underestimated, under-recognized, under-treated and linked to anxiety and depression (37, 39).

Most participants experienced pimples/acne compared to their pre-adolescent ages. All participants acknowledged that pimples/acne were natural adolescent experiences as they approach adulthood. The current findings confirm earlier reports that pimples/acne are very common problems that affect about 85% of young women aged 12–25 years worldwide with 20% of teenagers experiencing moderate to severe pimple/acne. This condition is said to have implications for their quality of life, self-esteem, personality, mood, and psychological disorders (40–42). Even though pimples/acne may not result in death, it “can scar people literally and psychologically” if not understood and well managed (42). Scientifically, pimples/acnes have been associated with increased production of androgens during adolescence but the current study has also linked them with the use of cosmetics and dietary choices such as diets high in proteins, sugar, fats and oils and refined grains usually associated with Western diets (43, 44). Additionally, the consumption of groundnut and oily foods in general has been implicated in this study.

Puberty, pregnancy and menopause have been reported as the life stages where women experience the most drastic fluctuations in their hormonal activities (45). Although part of the normal changes of puberty, these symptoms, if not well addressed and managed, may result in temporary and, at times, long-term physical and psychosocial problems. Persistent occurrence of these symptoms would, however, require medical examination and hormonal testing to determine their levels and the specific hormones involved (46). It would, therefore, be inappropriate to conclude that participants in this study experience hormonal imbalance without any medical assessment. It has been shown that many hormonal-related symptoms may be caused by other health conditions including endometriosis—a chronic disease linked with severe menstrual and pelvic pain, fatigue, infertility, depression and anxiety, affecting 10% of women—and not necessarily the result of hormonal imbalance (46).

### Management of perceived symptoms of hormonal imbalance in adolescent girls

Adolescent girls in this study may have found it more appropriate and much easier communicating their experiences with other female relatives since the latter were likely to have experienced similar symptoms. This finding has been reported elsewhere (28). Other adolescent girls consulted hormonal-related books, drugstore operators and school health prefects/nurses for advice. The ability to read and write, enhanced by formal education, increases people's access to information and this could explain why some adolescents resorted to hormonal-related books. In Ghana, sickbay facilities are available in some schools and manned by senior students, nurses and, in a few cases, doctors as reported in a previous study (47). Most boarding school participants in the study sought care at these sickbays when experiencing menstrual-related pains and headaches. Adolescents, however, kept symptoms of irregularity of menses and pimples to themselves apparently because they associated them with normal adolescent developmental process. They may also find menstrual irregularities a difficult topic to discuss as reported in an earlier study (14).

Drugstores appear to be more common and accessible in most communities and, hence, very convenient for adolescent girls to walk to for advice when they experience these symptoms. Use and abuse of over-the-counter medication by adolescents have been reported widely as becoming rampant health and safety concerns in both developed and developing countries (48). Easy access, inadequate knowledge of appropriate use, false perceptions of safety, social influences and lack of parental monitoring of adolescents' drug use may account for this practice. Using painkillers in managing all manner of pains including hormonal-related ones is common and reported by several studies (48–50). Adolescents may not consider their menstrual-related symptoms as serious health issues that need expert diagnosis and treatment as some of them perceived their symptoms as part of the adolescent growth process and, hence, natural.

Managing their symptoms through dietary modification, physical activity, and personal hygiene aligns with recommendations by experts for managing hormonal-related imbalances (51). Avoiding sugar-sweetened beverages, fatty and oily foods and oily cosmetics as against drinking lots of water, engaging in physical exercises, sleeping and resting when experiencing menstrual cramps has been reported in other studies (52, 53). Much as participants in the current study deserve commendation for adopting lifestyle behavioural changes to manage their symptoms, a few perceptions and practices with potential negative implications for their dietary quality and health were observed. Associating groundnut with pimples/acne and, therefore, avoiding its consumption should be of concern. Adolescent girls need to be educated on the nutritional and sustainability benefits of groundnut. Dietary guidelines all over the world are encouraging the consumption of more plant-based proteins as a sustainability measure to help save planet earth (54); however, if our future mothers are associating nutritious/healthy plant-based foods with negative health outcomes, then, one needs to be concerned. Consuming foods rich in fatty oils in moderation is highly recommended (54–56). The use of herbal concoctions reported by a few participants must be another matter of concern to all. The use of herbal agents in managing some health conditions is common globally with about 80% of people in developing countries depending on them as primary health care measures. Even though the efficacy of some of these agents has been proven, many remain untested with implications for poor health outcomes (57). Easy and unrestricted access, financial difficulties, cultural acceptability, and a belief in their potency may account for their popularity.

### Strengths and limitations of the study

The sample was drawn from rural and urban locations in northern and southern Ghana to enable an assessment of participants' knowledge and perceptions of hormonal imbalance to inform tailored interventions. Despite its geographical representation, inclusion of only 2 out of 16 administrative regions, 2 out 216 local government districts and only in-school adolescent girls make the findings not generalizable to the entire national population. Inclusion of other relevant categories of people such as out-of-school adolescent girls, parents/caretakers, teachers, and healthcare providers could have enriched the findings with their added perspectives. Self-reporting, an approach used in this study, may constitute a source of error/bias since the tendency to either over or under report experiences is a natural human frailty.

## Conclusion

In-school adolescent girls at the three levels of education in Ghana have differential knowledge of hormonal imbalance. Girls at all three levels experience some of the symptoms perceived to be associated with hormonal imbalance. Most of the girls adopt lifestyle behaviours including dietary measures, physical activities and practices of good hygiene to manage their symptoms even though some practice self-medication.

Ghana Education Service should expand its programmes including the School Health Education Programme (SHEP) to include adolescent-related hormonal changes and other issues of girls' reproductive health. There is the need for a nation-wide clinical study of the hormonal status among adolescent girls and its prevalence/magnitude. All stakeholder groups including parents/guardians, teachers/school authorities, healthcare providers, community and religious leaders and the adolescent girls themselves should be involved in action against the practice of self-medication among some adolescent girls to manage their perceived symptoms of hormonal imbalance.

## Data Availability

The raw data supporting the conclusions of this article will be made available by the authors, without undue reservation.
